# Phase 2 trial of palbociclib and ganitumab in patients with relapsed Ewing sarcoma

**DOI:** 10.1002/cam4.6208

**Published:** 2023-06-12

**Authors:** David S. Shulman, Priscilla Merriam, Edwin Choy, Lillian M. Guenther, Kerri L. Cavanaugh, Pei‐Chi Kao, Andrew Posner, Ketki Bhushan, Grace Fairchild, Emma Barker, Kelly Klega, Kimberly Stegmaier, Brian D. Crompton, Wendy B. London, Steven G. DuBois

**Affiliations:** ^1^ Dana‐Farber/Boston Children's Cancer and Blood Disorders Center and Harvard Medical School Boston Massachusetts USA; ^2^ Dana‐Farber Cancer Institute and Harvard Medical School Boston Massachusetts USA; ^3^ Massachusetts General Hospital Massachusetts General Hospital Cancer Center Boston Massachusetts USA; ^4^ St. Jude Children's Research Hospital Memphis Tennessee USA

**Keywords:** CDK4/6, Ewing sarcoma, ganitumab, IGF‐1R, palbociclib

## Abstract

**Background:**

Ewing sarcoma (EWS) is an aggressive sarcoma with few treatment options for patients with relapsed disease. Cyclin‐dependent kinase 4 (CDK4) is a genomic vulnerability in EWS that is synergistic with IGF‐1R inhibition in preclinical studies. We present the results of a phase 2 study combining palbociclib (CDK4/6 inhibitor) with ganitumab (IGF‐1R monoclonal antibody) for patients with relapsed EWS.

**Patients and Methods:**

This open‐label, non‐randomized, phase 2 trial enrolled patients ≥12 years with relapsed EWS. All patients had molecular confirmation of EWS and RECIST measurable disease. Patients initially received palbociclib 125 mg orally on Days 1–21 and ganitumab 18 mg/kg intravenously on Days 1 and 15 of a 28‐day cycle. The primary endpoints were objective response (complete or partial) per RECIST and toxicity by CTCAE. An exact one‐stage design required ≥4 responders out of 15 to evaluate an alternative hypothesis of 40% response rate against a null of 10%. The study was closed following enrollment of the 10th patient due to discontinuation of ganitumab supply.

**Results:**

Ten evaluable patients enrolled [median age 25.7 years (range 12.3–40.1)]. The median duration of therapy was 2.5 months (range 0.9–10.8). There were no complete or partial responders. Three of 10 patients had stable disease for >4 cycles and 2 had stable disease at completion of planned therapy or study closure. Six‐month progression‐free survival was 30% (95% CI 1.6%–58.4%). Two patients had cycle 1 hematologic dose‐limiting toxicities (DLTs) triggering palbociclib dose reduction to 100 mg daily for 21 days. Two subsequent patients had cycle 1 hematologic DLTs at the reduced dose. Eighty percent of patients had grade 3/4 AEs, including neutropenia (*n* = 8), white blood cell decreased (*n* = 7), and thrombocytopenia (*n* = 5). Serum total IGF‐1 significantly increased (*p* = 0.013) and ctDNA decreased during the first cycle.

**Conclusions:**

This combination lacks adequate therapeutic activity for further study, though a subset of patients had prolonged stable disease.

## 1 INTRODUCTION

Ewing sarcoma (EWS) is a rare, aggressive malignancy of bone and soft tissue affecting adolescents and young adults.^1^ Serial cooperative group studies have improved outcomes for patients with localized EWS to 78% at 5 years.^2^ Improvements in outcomes have largely come through intensification of conventional chemotherapy and improved local control strategies. Outcomes for patients with metastatic or relapsed disease remain poor.^3^, ^4^, ^5^, ^6^, ^7^, ^8^ Novel targeted therapies are needed for this patient population given that current therapy has neared maximal intensity and has provided minimal additional benefits in outcomes for patients with metastatic disease.

Several lines of evidence have suggested that IGF‐1R signaling is important in EWS. EWS cells are known to have high IGF‐1R expression and multiple early *in vivo* and *in vitro* studies have demonstrated sensitivity to IGF‐1R inhibitors.^9^, ^10^, ^11^, ^12^, ^13^, ^14^, ^15^, ^16^, ^17^ Clinical trials have demonstrated that approximately 10% of patients with relapsed disease respond to IGF‐1R monoclonal antibodies.^18^, ^19^, ^20^, ^21^ Following these studies, the IGF‐1R inhibitor ganitumab was tested in combination with standard frontline chemotherapy (vincristine/doxorubicin/cyclophosphamide alternating with ifosfamide/etoposide) in the phase 3 clinical trial AEWS1221 for patients with newly diagnosed metastatic EWS. The addition of ganitumab to conventional chemotherapy did not improve outcomes.^8^

Cyclin‐dependent kinase 4 (CDK4) has been implicated as a genomic vulnerability in EWS. EWS harbors a superenhancer at the cyclin D1 (*CCND1*) locus.^22^ Further genome‐wide screens confirmed CDK4 as a vulnerability in EWS. Based on these findings, a chemical library screen identified IGF‐1R as a synergistic target. In parallel, a genome‐scale open reading frame screen identified overexpression of IGF‐1R as a resistance mechanism to CDK4/6 inhibition. These findings were confirmed with *in vitro* and *in vivo* experiments.^23^

Given these preclinical findings, we designed a phase 2 clinical trial of the novel–novel combination of palbociclib (CDK4/6 inhibitor) and ganitumab (IGF‐1R inhibitor) for patients with relapsed or refractory EWS. The primary objectives were to assess the overall response rate to this combination and to assess toxicity in this patient population.

## 2 PATIENTS AND METHODS

### 2.1 Study design and participants

This was an investigator‐initiated nonrandomized phase 2 clinical trial of palbociclib and ganitumab in patients with relapsed or refractory EWS (NCT04129151). This study was conducted at a single center including pediatric and adult patients.

Participants were initially required to be ≥12 years and ≤ 50 years of age at study entry, and later any age ≥ 12 years following study amendment. A Karnofsky performance status of ≥50% for patients ≥16 years of age and Lansky performance status of ≥50% for participants <16 years of age was required. All participants were required to have RECIST v1.1 measurable disease.^24^ A histologic diagnosis of EWS with molecular confirmation of an *EWSR1* or *FUS* (also known as *TLS*) translocation was required. If the translocation partner was known, it was required to be an ETS family partner (e.g., *FLI1* or *ERG*). Standard washout periods and organ function parameters were utilized. Prior treatment with a CDK4/6 inhibitor was not allowed. Prior therapy with an IGF‐1R inhibitor was allowed if the patient did not have relapse or progression on that therapy. Participants with a prior history of pneumonitis were excluded given report of pneumonitis in patients who had received thoracic radiation and ganitumab on AEWS1221. Given the necessity of intact Rb‐mediated cell cycle progression to sustain response to CDK4/6 inhibitors, participants with a known *RB1* mutation or deletion were excluded.^25^

### 2.2 Study procedures

Participants initially received palbociclib 125 mg by mouth daily for 21 days and ganitumab 18 mg/kg intravenously on Days 1 and 15 in 28‐day cycles. Standard hematologic requirements were used as starting criteria. Participants were permitted to remain on therapy for up to 12 cycles in the absence of meeting off‐therapy criteria. Disease was assessed at baseline, during cycles 2, 4, 6, 8, 10, and 12 and at end of treatment.

### 2.3 Endpoints

The primary objective was to estimate the objective response rate. Objective response was assessed using RECIST 1.1. A patient was considered evaluable if they had received at least one dose of palbociclib or ganitumab and had at least one follow‐up disease evaluation of their RECIST measurable disease or evidence of clinical progression.

Assessment of toxicity according to the CTCAE v5.0 was another primary objective. Hematologic dose‐limiting toxicity (DLT) criteria included grade 4 thrombocytopenia or grade 4 neutropenia of any duration in the absence of bone marrow disease; or grade 3 or higher febrile neutropenia with or without documentation of infection; or grade 3 thrombocytopenia in association with a grade 2 or higher bleeding episode; or delay in the start of a subsequent cycle by >14 days due to thrombocytopenia or neutropenia in a participant without bone marrow disease.

Progression‐free survival (PFS; event defined as first occurrence of relapse, progression, or death from any cause) and overall survival (OS; event defined as death from any cause) were secondary efficacy endpoints, with patients censored at last follow‐up in the absence of an event.

### 2.4 Biomarker assessments

Serum IGF‐related proteins were assessed as an exploratory pharmacodynamic endpoint to demonstrate on‐target activity of ganitumab. Peripheral blood was collected prior to cycles 1, 2, and 4 in serum separator tubes. Serum was isolated, frozen, and batch shipped to Ansh Labs for testing using enzyme linked immunoassays for total IGF‐1, free IGF‐1, IGF‐2, and IGFBP‐3.

Circulating tumor DNA (ctDNA) was also assessed as an exploratory aim. Samples were collected at baseline, on Day 15 of cycle 1, and prior to cycles 2, 3, 5, 7, 9, 11, and at end of therapy. All samples were drawn into EDTA or CellSave tubes and processed same day in this single‐center study. Samples were sequenced at the Broad Institute, Cambridge, MA. Total cell‐free DNA was extracted from plasma using QIAmp Circulating Nucleic Acid Kit (Qiagen). Libraries were prepped using a KAPA Hyper Prep Kit (Kapa Biosystems) with manual barcoded adapter ligation. Samples underwent ultra‐low‐pass whole‐genome sequencing (ULP‐WGS at 0.1–0.5× coverage) and analyzed on the Broad's ichorCNA algorithm for ctDNA quantification. All samples then underwent analysis with TranSS‐Seq, a custom hybrid‐capture assay. Sequencing libraries were enriched with a Twist Bioscience Hybrid Capture Kit with a validated custom bait set targeting intronic regions of genes commonly involved in sarcoma translocations, including *EWSR1* and *FUS*.^26^ Fusions were identified either from sequencing of patient tumor samples, or from ctDNA using fusion callers, SvABA and BreaKmer. ctDNA was quantified by comparing the translocation reads to wild‐type reads with the following formula (%ctDNA = *T*/[([*W* − *T*]/2) + T], where *T* is the number of translocation reads and *W* is the number of wild‐type reads).

### 2.5 Statistical analysis

The response rate was defined as the proportion of responders among response evaluable patients. Per the Rule of Three, if there are no responders in a sample with *n* subjects, the interval from 0 to 3/*n* is an approximate 95% confidence interval (CI) for the response rate.^27^ The study design used a one‐stage exact rule with *n* = 15 planned evaluable patients. A null response rate of 10% was selected, which reflects an estimated response rate to IGF‐1R monotherapy for patients with relapsed/refractory EWS pooled from prior trials. The alternative hypothesis was that 40% or more patients would respond to the combination of palbociclib and ganitumab. If the number of responders was four or more, the hypothesis that the true response rate was less than or equal to 10% could be rejected with a type 1 error rate of 0.056. If the number of responders was three or fewer, the hypothesis that the response rate was 40% or greater could be rejected with 91% power.

Given this combination had not previously been tested in patients with relapsed and refractory EWS, safety stopping rules were used. Two toxicity monitoring rules were used in the trial: one from the original version of the protocol, and another in a protocol amendment required when the original stopping rule was triggered. In the original rule, toxicity was monitored using a Simon's two‐stage design, which required stopping if two or more patients out of nine treated, or three or more patients out of 15 treated, had a cycle 1 DLT. The operating characteristics of this rule were: a null hypothesis that the cycle 1 DLT rate is >30% versus the alternative that the cycle 1 DLT is ≤5%, an expected sample size of 10, a probability of early termination of 0.804 under the null hypothesis, power of 92%, and type 1 error of 9.5%. The revised stopping rule (applied to subsequent patients treated at a lower dose level) was an exact one‐stage rule which required stopping if three or more patients out of 12 treated had a cycle 1 DLT. The operating characteristics of this rule were: a null hypothesis that the cycle 1 DLT rate is ≥39% versus the alternative that the cycle 1 DLT is ≤9%, power of 91.3%, and type 1 error of 9.5%.

Analyses of demographic, clinical, safety, and ctDNA data were summarized descriptively. Paired *t*‐tests were used to compare intrapatient changes in IGF‐related proteins. Time‐to‐event data were analyzed using Kaplan–Meier methods, with 95% confidence intervals on PFS and OS point estimates calculated according to Greenwood. The changes in pharmacodynamic biomarker levels, from baseline to cycle 2, were tested using a two‐sided paired *t*‐test. There were no corrections made for multiple comparisons.

## 3 RESULTS

### 3.1 Patient characteristics

A total of 10 patients enrolled between December 2019 and August 2021. All 10 patients received at least one dose of study drug and were included in the primary efficacy and toxicity analyses. The study was closed in December 2021 prior to completion of accrual due to unanticipated inability to extend the expiration of the final ganitumab stock without planned further production. No unplanned analyses were conducted prior to this unanticipated early closure due to lack of available drug.

Of the 10 patients, 7 were male and the median age at enrollment was 25.7 years (range 12.3–40.1; Table 1). All patients had a histologic diagnosis of EWS and an identified *EWSR1* fusion. No patients had received prior IGF‐1R directed therapy.

**TABLE 1:** Characteristics of study participants with relapsed Ewing sarcoma (*n* = 10).

Characteristics	*n* (%)
Age at enrollment—median (range)	25.7 years (12.3–40.1)
Race	
White	8 (80%)
More than one race	2 (20%)
Ethnicity	
Non‐Hispanic	10 (100%)
Male	7 (70%)
Stage at diagnosis	
Localized	4 (40%)
Metastatic	6 (60%)
Primary site at diagnosis	
Bone	8 (80%)
Soft tissue	2 (20%)
Primary site at diagnosis	
Femur	3 (30%)
Iliac	2 (20%)
Gluteal soft tissue	2 (20%)
Scapula	1 (10%)
Tibia	1 (10%)
Thoracic vertebra	1 (10%)
Translocation	
*EWSR1/FLI1*	5 (50%)
*EWSR1*‐translocation by FISH	5 (50%)
Prior IGF‐1R therapy	
No	10 (100%)
Yes	0 (0%)

### 3.2 Toxicity and safety

Toxicity data are presented in Table 2. Two patients experienced cycle 1 hematologic DLTs, triggering the original toxicity stopping rule. The palbociclib starting dose was reduced from 125 to 100 mg daily for 21 days for these and subsequent patients. The ganitumab dose was not changed. A new toxicity stopping rule was applied to patients treated at the lower dose of palbociclib. Among the remaining 8 patients, two had cycle 1 hematologic DLTs at the 100 mg dose, with one patient requiring a dose reduction and the other coming off‐treatment due to disease progression prior to resuming therapy. These two DLTs did not trigger the subsequent toxicity stopping rule.

**TABLE 2:** Grade 2 and greater toxicities related (possibly, probably, or definitely) to palbociclib and/or ganitumab therapy (*n* = 10).

Toxicity type	Maximum toxicity grade *n* (%)		
	Grade 2	Grade 3	Grade 4
Hematologic toxicity			
Anemia	2 (20%)	0 (0%)	0 (0%)
Lymphocyte count decreased	3 (30%)	0 (0%)	1 (10%)
Neutrophil count decreased	1 (10%)	5 (50%)	3 (30%)
Platelet count decreased	2 (20%)	4 (40%)	1 (10%)
White blood cell decreased	1 (10%)	7 (70%)	0 (0%)
Non‐hematologic toxicity			
Fatigue	1 (10%)	0 (0%)	0 (0%)
Infusion related reaction	1 (10%)	0 (0%)	0 (0%)
Hypophosphatemia	1 (10%)	0 (0%)	0 (0%)
Metabolism and nutrition disorders—vitamin D deficiency	1 (10%)	0 (0%)	0 (0%)
Hypertension	0 (0%)	1 (10%)	0 (0%)

*Note*: Adverse events were reported at the maximum grade per type per patient, regardless of cycle in which the adverse events were observed.

Grade 3/4 AEs were observed in 80% of patients. The most common grade 3/4 AEs were neutropenia (8/10 patients), white blood cell count decreased (7/10 patients), and thrombocytopenia (5/10 patients). No patients were removed from the study due to toxicity.

### 3.3 Efficacy

**FIGURE 1:**
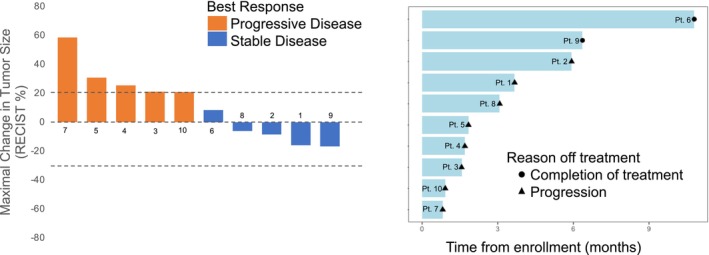
Response and outcome data. Waterfall plot (A) and swimmers plot (B) for the 10 patients treated with palbociclib and ganitumab.

Although minor tumor regressions were seen in four patients, no RECIST‐defined partial responses were observed (Figure 1A; approximate 95% CI on the response rate of 0/10 was 0%–30%). The exact one‐stage rule required four or more responders out of 15. With 10 patients enrolled at the time of ganitumab expiration, and no patients with a RECIST‐defined response, the study team concluded that it was unlikely that four responders would be observed in the next five patients enrolled.

**FIGURE 2:**
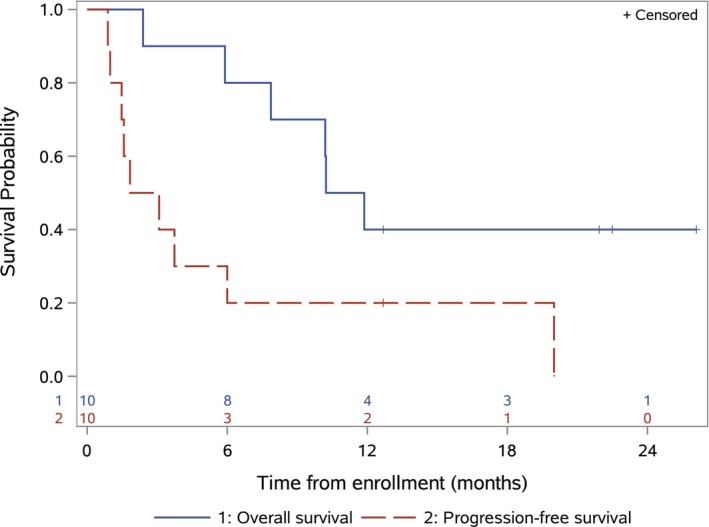
Progression‐free survival and overall survival. Progression‐free survival from date of enrollment (red dashed line) and overall survival from date of enrollment (blue solid line) for the 10 patients treated with palbociclib and ganitumab. Tick marks indicate patients who were censored.

The median duration of therapy was 2.5 months (range 0.9–10.8) and five patients had stable disease as best response (Figure 1B). One patient completed all 12 planned cycles of protocol therapy with stable disease and another patient remained on therapy at cycle 6 with stable disease at time of early study closure. The 6‐month PFS and OS estimates were 30% (95% CI 1.6–58.4) and 80% (95% CI 55.1–100), respectively (Figure 2).

### 3.4 Biomarkers

**FIGURE 3:**
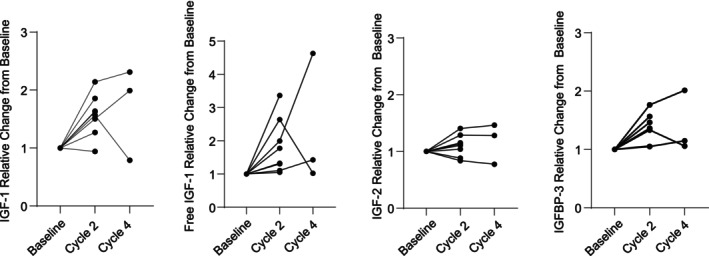
Relative changes in IGF‐1‐related proteins. Relative changes from baseline to cycle 2 day 1 and cycle 4 day 1 in total IGF‐1 (A), free IGF‐1 (B), IGF‐2 (C), and IGFBP‐3 (D). Baseline data were unavailable for two patients due to COVID‐19 restrictions.

Two patients did not have baseline and cycle 2 IGF‐related proteins levels collected due to study staff restrictions during the COVID‐19 pandemic, leaving eight patients evaluable for relative changes in serum IGF‐related proteins (Figure 3). There were statistically significant intra‐patient increases from baseline to start of cycle 2 in total IGF‐1, free IGF‐1, and IGFBP‐3, but not IGF‐2 (*p* = 0.013, *p* = 0.021, *p* = 0.02, *p* = 0.19, respectively).

**FIGURE 4:**
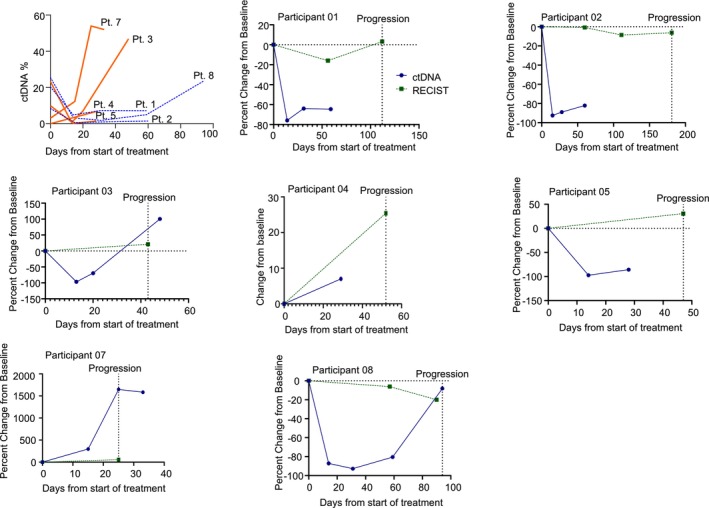
Circulating tumor DNA and RECIST measurements for all patients with detectable ctDNA. (A) Absolute ctDNA levels over time for all participants with detectable ctDNA. Patients with best response as progressive disease have orange solid lines and patients with best response as stable disease have a blue dashed lines. (B–H) are individual patient ctDNA and RESIST measurements. In green dashed is RECIST percent change from baseline and in blue solid is percent change in ctDNA from baseline. For participant 4 (E), ctDNA was not detectable at baseline so we show absolute change from baseline in %ctDNA and relative change from baseline for RECIST.

Circulating tumor DNA levels stratified by best response are shown in Figure 4A. Individual patient ctDNA levels and RECIST measurements are shown in Figure 4B–H. Patient 6 did not have ctDNA samples drawn until cycle 3 due to COVID‐19 study staff restrictions. Three patients did not have a detectable fusion in their plasma above the known sensitivity of 1% ctDNA using our NGS‐assay. All patients with a rise in ctDNA level at Day 15 had disease progression at their first disease assessment. All patients with stable disease as best response had declines in their ctDNA levels at Day 15. Three patients (1, 2, and 5) had persistent declines in their ctDNA levels and ultimate progression. However, they did not have ctDNA draws at time of radiographic progression. Patient 8 had a substantial decline in ctDNA content early in treatment at multiple timepoints prior to a rise in ctDNA at time of radiographic disease progression Figure 4H.

## 4 DISCUSSION

The primary objectives of this study were to determine the objective response rate to palbociclib and ganitumab in patients with relapsed EWS and to determine the safety of giving this treatment combination. No patients met the RECIST definition of objective response. The combination therapy, which was based on palbociclib dosing established in patients with breast cancer, required dose reduction in this heavily pretreated population. While two patients had prolonged stable disease, without further work to define biomarkers of sensitivity, the combination of IGF‐1R and CDK4/6 inhibition does not warrant further testing in this population.

Numerous preclinical studies have identified the IGF‐1 pathway as a potential vulnerability in EWS. Multiple prior early phase studies of IGF‐1R inhibitors in solid tumors, as well as in EWS specifically, have demonstrated an approximately 10% response rate. A recent randomized phase 3 study compared interval compressed vincristine/doxorucibin/cyclophosphamide and ifosfamide/etoposide with and without ganitumab and demonstrated no benefit from the addition of ganitumab. In the context of lack of benefit from combining IGF‐1R inhibition with conventional chemotherapy in metastatic EWS, the current study was developed after additional preclinical data demonstrated synergistic effects between CDK4/6 inhibition and IGF‐1R inhibition. Our study demonstrated a 6‐month PFS of 30%, which compares favorably with a recent analysis of seven COG phase 2 studies in EWS, which demonstrated a 6‐month PFS of 13%.^28^ This PFS also compares favorably to prior trials of IGF‐1R inhibitors that demonstrated 6‐month PFS that ranged from 9% to 40%.^17^, ^20^ Two patients on our study had prolonged stable disease and were removed from the study at end of planned therapy and study closure. Neither of these two patients had detectable ctDNA by our conventional NGS assay and a more sensitive ctDNA analysis is in development. Interestingly, 5 of 7 patients had a decline in their ctDNA levels by Day 15 of cycle 1 indicating that some patients may have had a transient benefit from this therapy. Three of these five patients had decreased tumor volume by RECIST at their first disease assessment. Our pharmacodynamic studies revealed intra‐patient elevations in IGF‐1 related proteins indicating on‐target IGF‐1R inhibition. While we did not utilize a specific pharmacodynamic marker for CDK4/6 inhibition, we started with FDA‐approved dosing of palbociclib and observed dose‐dependent neutropenia. We conclude that the lack of robust activity observed in this cohort is not related to lack of on‐target inhibition of IGF‐1R and CDK4/6.

The reasons for the lack of response in this study are not clear. The most likely fundamental explanation is that the dependency on the combination of CDK4 and IGF‐1R activity in laboratory models is not present in patients with advanced EWS. This could be due to redundant signaling in both the CDK4 and IGF‐1R pathways, or only heterogeneous expression of the IGF‐1R receptor in advanced EWS. In colorectal cancer, nuclear localization of IGF‐1R increased in metastatic tumors compared to primary tumors and was associated with chemotherapy and IGF‐1R targeted therapy resistance.^29^ Similarly, these tumors may acquire CDK4/6 inhibitor resistance in multiple ways including activation of alternative pathways such as other CDKs or *RB1* loss.^30^

Our study advances the use of ctDNA in clinical trials of EWS and other fusion‐positive sarcomas. Multiple studies have previously demonstrated the feasibility of quantifying ctDNA through fusion detection in EWS and that baseline ctDNA levels are prognostic in patients with newly diagnosed disease.^31^, ^32^, ^33^, ^34^ Our study demonstrates that ctDNA levels may be an early marker of response on a clinical trial, but early changes may not be associated with prolonged clinical responses. Further, given that ctDNA is present throughout treatment for most patients, future studies could include deeper sequencing of ctDNA for analysis of tumor evolution in this heavily treatment‐resistant population.

The initial planned palbociclib dosing in our study was established based on previously used dosing in patients with advanced breast cancer. Two patients experienced dose‐limiting neutropenia at that dose, triggering predefined study stopping rules. Given this toxicity was thought most likely related to CDK4/6 inhibition, the starting dose of palbociclib was reduced to 100 mg for 21 days of a 28‐day cycle. This revised dosing was tolerable for most patients. This toxicity experience highlights the importance of re‐assessing tolerability of an established dosing strategy being applied to a new population.

Our study was terminated early due to unanticipated inability to extend the expiration of the remaining available supply of ganitumab. At time of closure, 0/10 patients had an objective response. It is a limitation that the study was closed early, and we were therefore unable to assess 15 evaluable patients. However, it is unlikely that we would have seen four objective responses in the remaining five patients. An additional limitation of our study was that multiple correlative samples could not be collected for two patients who enrolled early in the COVID‐19 pandemic. Nevertheless, one of those patients was able to safely remain on therapy for a full 12 cycles of treatment with stable disease and minimal toxicity. This speaks to the ability of study teams to ensure that clinical trials remained available for patients during the pandemic. Most notably, this impacted baseline ctDNA collection for the patient with the most prolonged stable disease. Finally, the ctDNA assays used in our study were customized for identifying EWS fusions. More extensive ctDNA sequencing to identify mechanisms of resistance (e.g., *RB1* alteration) may have improved our understanding of lack of efficacy of this combination but was beyond the scope and funding of this study.

IGF‐1R inhibitors have now been tested as monotherapy, in combination with standard chemotherapy and now in combination with another targeted therapy. Despite strong preclinical evidence, there remains minimal clinical activity of these agents for patients with EWS. Since the start of this study, teprotumumab was approved for the treatment of thyroid eye disease and remains the only FDA‐approved IGF‐1R inhibitor.^35^, ^36^ For patients with EWS, further clinical evaluation of IGF‐1R directed therapies seems unwarranted without further understanding of the optimal approach to targeting IGF‐1R and which patients would be most likely to benefit from this strategy.

## AUTHOR CONTRIBUTIONS

**David S Shulman:** Conceptualization (equal); data curation (equal); formal analysis (equal); investigation (equal); methodology (equal); project administration (lead); supervision (equal); writing – original draft (lead); writing – review and editing (lead). **Priscilla Merriam:** Investigation (equal); methodology (equal); supervision (equal); writing – original draft (equal); writing – review and editing (equal). **Edwin Choy:** Methodology (equal); supervision (equal); writing – original draft (equal); writing – review and editing (equal). **Lillian Guenther:** Conceptualization (equal); formal analysis (equal); methodology (equal); writing – original draft (equal); writing – review and editing (equal). **Kerri Cavanaugh:** Investigation (equal); supervision (equal); writing – review and editing (equal). **Pei‐Chi Kao:** Conceptualization (equal); data curation (equal); formal analysis (equal); investigation (equal); methodology (equal); visualization (equal); writing – original draft (equal); writing – review and editing (equal). **Andrew Posner:** Data curation (equal); formal analysis (equal); investigation (equal); project administration (equal); writing – original draft (equal); writing – review and editing (equal). **Ketki Bhushan:** Investigation (equal); project administration (equal); writing – review and editing (equal). **Grace Fairchild:** Data curation (equal); investigation (equal); project administration (equal); writing – review and editing (equal). **Emma Barker:** Investigation (equal); supervision (equal); writing – review and editing (equal). **Kelly Klega:** Data curation (equal); formal analysis (equal); investigation (equal); methodology (equal); writing – original draft (equal); writing – review and editing (equal). **Kimberly Stegmaier:** Conceptualization (equal); investigation (equal); methodology (equal); writing – original draft (equal); writing – review and editing (equal). **Brian Crompton:** Conceptualization (equal); data curation (equal); formal analysis (equal); methodology (equal); writing – original draft (equal); writing – review and editing (equal). **Wendy London:** Conceptualization (equal); data curation (equal); formal analysis (equal); investigation (equal); methodology (equal); writing – original draft (equal); writing – review and editing (equal). **Steven DuBois:** Conceptualization (lead); data curation (equal); formal analysis (equal); funding acquisition (equal); investigation (equal); methodology (equal); project administration (equal); supervision (equal); writing – original draft (equal); writing – review and editing (equal).

## FUNDING INFORMATION

Ganitumab was supplied by ImmunityBio. Palbociclib was provided by Pfizer. Funding for the support of the trial was provided by 1 Million 4 Anna Foundation, Carson Sarcoma Foundation, Teaming up to Fight Childhood Cancer, ChemoWarrior: the eli sidler foundation, i‐ROK Foundation, Rutledge Cancer Foundation, and Alan B. Slifka Foundation. In addition, support was provided by Alex's Lemonade Stand Foundation (DSS, WBL, PK, and SGD), the Harvard Catalyst KL2/CMeRIT program (DSS), The Boston Children's Office of Faculty Development BTREC/CTREC (DSS) and Conquer Cancer the ASCO Foundation (DSS). LMG received support from the Damon Runyon Cancer Research Foundation and the Rally Foundation for Childhood Cancer Research.

## CONFLICT OF INTEREST STATEMENT

DSS reports consulting fees from Boehringer Ingelheim and Merlin Biotech. SGD reports consulting fees from Amgen, Bayer, and Jazz and travel expenses from Loxo Oncology, Roche, and Salarius. KS receives grant funding from the DFCI/Novartis Drug Discovery Program and from KronosBio, is a member of the SAB and has stock options with Auron Therapeutics and has consulted for AstraZeneca. WBL reports prior consulting fees from Merck Sharp & Dohme Corp, ArQule Inc, and Jubilant DraxImage Inc for service on Data Safety Monitoring Boards, from Y‐mAbs Therapeutics, Inc for service on their Scientific Advisory Board, and as a consultant to Healthcasts. EC receives research support from Amgen, Astra‐Zeneca, Exelixis, GSK, Iterion, Novartis, Merck, and Mirati, and has been paid for Advisory Board participation for Bayer, Adaptimmune, and Epizyme.

## ETHICS STATEMENT

This trial was approved by the Dana‐Farber/Harvard Cancer Center Institutional Review Board. Informed consent was reviewed and signed by all patients prior to study entry.

## Data Availability

The clinical data generated from this study are included in the article. Correlative biology data can be provided by the authors upon reasonable request.
